# Trust in the provider and accurate self-reported PrEP adherence among adolescent girls and young women in South Africa and Zimbabwe: HPTN 082 study

**DOI:** 10.1186/s12905-023-02418-9

**Published:** 2023-05-19

**Authors:** Geetha Beauchamp, Deborah Donnell, Sybil Hosek, Peter L. Anderson, Kwun C. G. Chan, Bonnie J. Dye, Nyaradzo Mgodi, Linda-Gail Bekker, Sinead Delany-Moretlwe, Connie Celum

**Affiliations:** 1 Department of Health Systems and Population Health, University of Washington, Seattle, WA USA; 2 Vaccine and Infectious Disease Division, Fred Hutchinson Cancer Research Center, 1100 Fairview Ave. N., Mail Stop M2-C200, WA 98109 Seattle, USA; 3 Department of Psychiatry, Stroger Hospital of Cook County, Chicago, IL USA; 4 Department of Pharmaceutical Sciences, University of Colorado-Anschutz Medical Campus, Aurora, CO USA; 5 Department of Biostatistics, University of Washington, Seattle, WA USA; 6 FHI 360, Durham, NC USA; 7 University of Zimbabwe Clinical Trials Research Centre, Harare, Zimbabwe; 8 The Desmond Tutu HIV Centre, University of Cape Town, Cape Town, South Africa; 9 Wits RHI, Faculty of Health Sciences, University of the Witwatersrand, Johannesburg, South Africa; 10 Departments of Global Health, Medicine, and Epidemiology, University of Washington, Seattle, WA USA

**Keywords:** Trust, Provider, Adherence, Pre-exposure prophylaxis, African adolescent girls and young women

## Abstract

**Background:**

Trust is an important cornerstone of patient-provider communication. Accurate reporting of pre-exposure prophylaxis (PrEP) adherence is vital for providers to determine who needs adherence support, especially adolescent girls and young women (AGYW) disproportionately affected by newly diagnosed HIV.

**Methods:**

This is a secondary analysis of the HPTN 082 open-label PrEP demonstration trial. From 2016–2018, 451 AGYW aged 16–25 years were enrolled in South Africa (Cape Town and Johannesburg) and Zimbabwe (Harare). PrEP was initiated by 427, and 354 (83%) had month three patient-reported adherence responses and intracellular tenofovir diphosphate (TFV-DP) measurements. The patient-reported adherence response to ‘In the past month, how often did you take the tablet?’ was dichotomized as ‘*high’* if the response was every day or most days, and ‘*low’* if some days or not many days or never. The biomarker marker evidence of adherence in dried blood spots was defined as ‘*high’* if TFV-DP ≥ 700, and *‘low’* if < 350 fmol/punch. We used multinomial logistic regression to examine if trust in the PrEP provider was associated with concordance between patient-reported adherence and intracellular tenofovir-diphosphate (TFV-DP).

**Results:**

AGYW who reported trust in their providers were almost four-fold (aOR 3.72, 95% CI 1.20–11.51) more likely to have concordant adherence (high self-reported adherence and high TFV-DP concentrations) compared to discordant non-adherence (high self-reported adherence and low TFV-DP concentrations).

**Conclusion:**

Education and training of providers to build trusting relationships with AGYW may lead to more accurate reporting of PrEP adherence. With accurate reporting, adequate support can be provided to bolster adherence.

**Trial registration:**

ClinicalTrials.gov Identifier: NCT02732730.

## Background

Of 1.7 million new HIV infections globally in 2019, 25% occurred in adolescent girls and young women (AGYW) aged 15–25 years in sub-Saharan Africa [1]. Pre-exposure prophylaxis (PrEP) is highly effective in preventing HIV acquisition when taken consistently [2–4]. Despite high initial uptake of PrEP by African adolescent girls and young women (AGYW) in demonstration studies, adherence declined within six months of starting PrEP [5–8]. Poor adherence to PrEP can lead to HIV acquisition. Consequently, an AGYW with HIV faces lifelong antiretroviral therapy (ART) to prevent AIDS and risk of transmission to sex partners and children [3, 9, 10].

The World Health Organization (WHO) recommends quarterly PrEP adherence monitoring for patients on PrEP [11]. The cost of biomarkers of PrEP adherence using plasma, an indicator of PrEP taken within the last 2–3 days, was approximately USD $50–150 per test. Intracellular tenofovir-diphosphate (TFV-DP) in dried blood spots (DBS), an indicator of average PrEP doses per week taken over the last 4–6 weeks, is about USD $200 per test. Because of DBS cost and burden of collection, patient-reported adherence is advantageous, as it is simple to ascertain by questionnaire, is inexpensive, and is a low-burden measure. In studies among adolescents in Africa and the United States (U.S.), patient-reported ART was significantly associated with viral load, suggesting that self-reported PrEP adherence may be used for clinical monitoring in the absence of biomarker assessment [12, 13]. However, self-reported PrEP adherence was substantially higher than biomarker concentrations of PrEP in several placebo-controlled PrEP clinical trials [14–16].

Trust is necessary for patients to believe that the health provider will act in their best interest in an inherently asymmetrical patient-provider relationship [17]. Hall et al. described that trust is at the core of the doctor-patient relationship, a defining characteristic that gives the relationship meaning, importance, and substance [18]. A literature review of qualitative studies on the role of trust in healthcare systems in sub-Saharan Africa found that trust between patients and providers was a critical element in fostering demand for health services, including HIV services [19]. Respect for individual patient needs and understanding of the patient’s values and beliefs are essential factors in providers acquiring trust and delivering quality healthcare, especially in the context of reproductive health and HIV-related health services in sub-Saharan Africa [20]. Cultural norms about adolescents’ sexual encounters outside of marriage contribute to provider bias and AGYW experiencing discriminatory care [21]. A youth-friendly, *nonjudgmental* environment that recognizes the dynamic nature of adolescents’ sexuality and self-autonomy will likely foster open communication between provider and patient to formulate patient-centric PrEP adherence strategies jointly [22, 23].

There is, however, limited research on the role of trust in the provider and PrEP adherence. Qualitative research conducted among women in sub-Saharan Africa in placebo-controlled efficacy trials and open-label PrEP demonstration projects revealed that community distrust of the study drug or the study team contributed to low adherence to PrEP [24–26]. Poor clinician attitude was identified as one reason for PrEP discontinuation in a recent qualitative study that examined PrEP discontinuation in participants who initiated PrEP in HIV comprehensive care centers in Kenya, [27]. Similarly, qualitative studies on preventing perinatal transmission services in Africa described that negative attitudes of care providers and disrespectful treatment of patients by providers resulted in abandoning ART [28]. In VOICE, a placebo-controlled efficacy trial of oral PrEP and vaginal tenofovir gel for HIV prevention among African women, trust in the researchers was recognized as a facilitator by women with biomarker evidence of adherence [29].We did not find published research that examined whether patients are likely to be transparent about their PrEP adherence if they have a trusting relationship with their provider.

In this study, we quantitatively evaluated the association between trust in PrEP providers (research clinicians and counselors) and the concordance between self-reported and biomarker PrEP adherence among AGYW from the HIV Prevention Trials Network (HPTN 082) study. We hypothesized that having a trusting relationship with the PrEP provider was associated with concordant adherence (high self-reported and high biomarker) and concordant non-adherence (low self-reported and low biomarker) and that a low level of trust in the PrEP provider was associated with discordant non-adherence (high self-reported and low biomarker). In addition, we hypothesized that the perception of greater likelihood of HIV acquisition was associated with PrEP adherence [30]. Hence, we examined if perception of greater likelihood of HIV acquisition was associated concordant adherence.

## Methods

### Study design and population

We conducted a secondary analysis of the HPTN 082 open-label PrEP demonstration trial, which was conducted in adolescent and youth-friendly research clinics among 451 AGYW (ages 16 to 25 years) without HIV between 2016 and 2018 in Cape Town and Johannesburg, South Africa; and Harare, Zimbabwe. To participate in the HPTN 082 trial, women needed to be literate, female at birth, ages 16–25 years, reported interest in using PrEP, had sex in the past month, scored ≥ 5 in the VOICE risk behavior questionnaire, had regular access to phone with short message service (SMS) functionality, be without hepatitis B, had normal renal function and not be pregnant [31]. All participants were offered daily oral tenofovir disoproxil fumarate (TDF, 300 mg) co-formulated with emtricitabine (FTC, 200 mg) as PrEP for one year. During enrollment and follow-up visits at months one and two and then quarterly for one year, participants received PrEP refills sufficient to last until the next visit. Participants who initially declined PrEP could initiate PrEP during follow-up. Participants who accepted PrEP were randomized 1:1 to the standard of care (SOC) adherence arm or enhanced adherence support arm. Participants in both arms received the following adherence support services: brief adherence counseling at monthly visits from months one through three and quarterly thereafter based on cognitive behavioral therapy. Participants also received a two-way weekly SMS reminder during the first three months. They were invited to participate voluntarily in monthly peer support adherence clubs, including discussions about factors that facilitated or hindered PrEP adherence. Participants assigned to the enhanced adherence arm also received retrospective semi-quantitative and motivational drug level feedback at months two and three based on intracellular TFV-DP levels in DBS samples taken at months one and two [32, 33].

### Data collection

Demographic data, including age, education, and housing status, were collected at enrollment. Patient’s self-reported data were collected using computer-assisted self-interviews (CASI). CASI data, including HIV likelihood perception, sexual behavior, alcohol use, substance use, and depressive symptoms, were collected at enrollment and then quarterly for up to 12 months. A brief version (10-items) of the Center for Epidemiologic Studies Depression Scale (CESD-10) was used to assess depressive symptoms [34, 35]. The question, ‘How would you describe your chances of getting HIV in the next year’ was dichotomized as *perceived HIV likelihood in the next year* if responses were small, moderate, great chance, and prefer not to answer [36]. Transaction sex was defined as having sex with a man in exchange for food, clothes, cosmetics, transportation, and items for children, and other items. ‘Prefer not to answer’ responses to condom use questions were categorized as condomless sex.

### Provider characteristics

Data on participant perceptions of provider characteristics indicators were collected at two time points using CASI:enrollment and month three. We used data from month three to asesses trust in the PrEP healthcare team. The three trust indicators were: i) I have a strong, trusting relationship with the study staff; ii) even when it is difficult, I let the study staff know if I have missed doses of my PrEP; and iii) I know how to contact the study doctor/nurse if I have problems or questions about PrEP. The responses at month three were assumed to reflect participants’ experiences with the PrEP providers in the previous three months. Responses were rated on a 5-point Likert-scale ranging from ‘strongly agree’ to strongly disagree’. They were dichotomized as *agree* if the responses were ‘agree or strongly agree’ and *disagree* if the responses were ‘neither agree/disagree, disagree, or strongly disagree’. Between 9 (3%) and 15 (6%) of the responses were ‘prefer not to answer’ for the three trust indicators described above, and they were categorized as *disagree*.

### PrEP adherence

DBS specimens were collected at months one and two and then quarterly for up to 12 months to assess TFV-DP drug concentrations. Tenofovir-diphosphate (TFV-DP) concentration in DBS was used as a biomarker indicator of adherence, which is considered an objective measure reflecting the average PrEP adherence over the past 4–6 weeks [37]. For AGYW, we defined DBS TFV-DP ≥ 700 fmol/punch as *high* adherence and DBS TFV-DP < 350 fmol/punch as *low* adherence. These thresholds correspond to ≥ 4 doses per week and < 2 doses per week, based on directly observed dosing studies in the U.S. [37]. These concentrations were selected because in the iPrEX open label extension study, the HIV risk reduction was estimated to be 100% (95%CI: 86%-100%) among men who have sex with men (MSM) who had TFV-DP concentrations associated with an average of four doses per week [38]. Concentration thresholds for DBS TFV-DP and efficacy have not yet been established for women, although no efficacy was observed in randomized clinical trials when adherence was low. Based on retrospective plasma tenofovir concentrations, only 25%-30% had taken PrEP in the prior week [39, 40]. To dichotomize adherence into high versus low adherence, women with DBS TFV-DP concentrations in the intermediate range (350–700 fmol/punch), which is associated with an average of 2–3 doses per week [37], were excluded from the analysis (*n* = 84).

Patient-reported adherence was collected for approximately the same timeframe as DBS samples for TFV-DP concentrations. The response to the question, ‘in the *past month*, how often did you take the tablet?’ was used for *patient-reported* PrEP adherence. Responses were rated on a 5-point Likert-scale and dichotomized as *high* if the participant responded, ‘every day or most days’, and *low* if the responses were ‘some days, not many days, or never’. Participants who responded, ‘prefer not to answer’ (*n* = 3) were categorized as *low* patient-reported adherence*.* We combined patient-reported and TFV-DP adherence biomarker data at the month three visit (same follow-up study visit as provider characteristics data) to define the outcome measure with three distinct levels of adherence: *concordant adherent* (high patient-reported and high TFV-DP concentrations), *concordant non-adherent* (low patient-reported adherence and low TFV-DP concentrations) and *discordant non-adherent* (high patient-reported adherence and low TFV-DP concentrations). A few AGYW (*n* = 5) under-reported (low patient-reported and high DBS TFV-DP concentrations) PrEP adherence and were excluded because of the small sample size.

### Statistical analysis

Baseline demographics, HIV likelihood perception, and behaviorally vulnerability to HIV were summarized using descriptive statistics stratified by concordant and discordant adherence groups. The Kruskal–Wallis test was used to test group differences for continuous variables, and the Pearson Chi-squared test was used for categorical variables. The log odds of the concordant adherent relative to the discordant non-adherent and log odds of the concordant non-adherent relative to the discordant non-adherent were modeled as a linear function of trust indicators, using multinomial logistic regression. The three included sites have similar HIV incidence, yet there may be different social and economic differences by site, therefore site [1] was the only covariate in univariate and multivariate analyses. A backward selection method was applied with multivariable multinomial logistic regression for trust indicators with *p*-values < 0.1 in the univariate analysis.

In a sensitivity analysis, we repeated the analysis with undetectable TFV-DP as *no-to-low* adherence to assess whether our findings were robust to changes in the definition of the discordant non-adherent. DBS TFV-DP below the lower limit of quantification indicates no evidence of PrEP use in the prior month. In addition, subgroup analysis of *high trust* and *low trust* in the provider was performed separately. For this, we used a t-test to compare the continuous measure of DBS TFV-DP concentration between those who reported taking pills ‘most days’ (every day/most days) with ‘not often’ (some days/not many days/never). All analyses were conducted using SAS version 9.4 (SAS Institute, Cary, NC, USA).

## Results

### Participant characteristics

Of the 427 AGYW who accepted PrEP in the HPTN 082 study, 381 (89%) completed the three-month visit. Both biomarker (DBS TFV-DP) and patient-reported adherence data were available for 354 (83%) participants. Eighty-three (23%) of the AGYW were ‘concordant adherent’; 56 (16%) were ‘concordant non-adherent’; 126 (36%) were ‘discordant non-adherent’; 5 (1%) were discordant adherent (not analyzed due to small number); 84 (24%) had TFV-DP concentrations in the intermediate range and were not categorized as concordant or discordant. Participant demographic characteristics were generally similar across all three groups: concordant adherent, concordant non-adherent, and discordant non-adherent. The median age of participants across the three groups was 21 years; over 50% had received secondary education or higher, and nearly 90% reported having a regular place to stay. At enrollment, almost 80% had a primary partner who was 3–5 years older, and approximately 50% of the partners lived with HIV or were of unknown HIV status (Table 1). Discordant non-adherent participants were more likely to have more than one sex partner (19%, *p* = 0.04) in the last three months than ‘concordant adherent’ (7%) or ‘concordant non-adherent’ (11%) participants. There was a non-significant higher HIV likelihood perception among the ‘concordant adherent’ (61%, *p* = 0.07) than both ‘discordant non-adherent’ (45%) and ‘concordant non-adherent’ (49%). Similarly, a higher percentage of the ‘concordant adherent’ reported partner financial support (63%, *p* = 0.06) compared to ‘discordant non-adherent’ (46%) and ‘concordant non-adherent’ (43%).

**Table 1:** Baseline characteristics of study participants

**Baseline Characteristics** N (%) or (median, IQR)	**Concordant Adherent** *N* = 83	**Discordant Non-adherent** *N* = 126	**Concordant Non-adherent** *N* = 56	***P*-value**^**a**^
Age, median (years)	21 (20,23)	21 (19,22)	20 (19,22)	0.15
Secondary education	49 (59%)	76 (60%)	30 (54%)	0.69
Regular place to stay	75 (90%)	111 (88%)	51 (91%)	0.66
At least a small chance of acquiring HIV in the next year^b^	51 (61%)	57 (45%)	28 (49%)	0.07
Alcoholic drinks past three months	59 (71%)	79 (63%)	37 (66%)	0.43
Has Primary Partner, last 3 months	75 (90%)	106 (84%)	47 (84%)	0.32
Primary partner living with HIV or unknown HIV status	37 (45%)	64 (51%)	33 (59%)	0.25
Age difference with primary partner, median (years)	4 (2,6)	5 (2,7)	3 (2,5)	0.19
Condomless vaginal sex, last month^c^	69 (83%)	100 (79%)	50 (89%)	0.29
Condomless anal sex, last month^c^	26 (31%)	58 (46%)	24 (43%)	0.10
Have more than one sex partner, last 3 months	6 (7%)	24 (19%)	6 (11%)	0.04
Had alcohol or drug before sex, last month	24 (29%)	34 (27%)	20 (36%)	0.50
Partner provides financial support	52 (63%)	58 (46%)	24 (43%)	0.06
Transactional sex, last 3 months^d^	22 (27%)	30 (24%)	12 (21%)	0.78
Sexually transmitted infection	36 (43%)	45 (36%)	29 (52%)	0.12
CES-D Depression score ≥ 10^e^	34 (41%)	64 (51%)	31 (55%)	0.20
Intimate partner violence in the past year	41 (49%)	61 (48%)	27 (48%)	0.99

^a^The median and interquartile range are provided for continuous variables; *p*-values are from the Kruskal Wallis test. Percentage and frequency are provided for categorical variable; *p*-values are from Pearson Chi-squared test

^b^‘How would you describe your chances of getting HIV in the next year’ was dichotomized as *at least a small chance* (small/ moderate, great chance and prefer not to answer) and *none* (no chance)

^c^‘Prefer not to answer’ responses were categorized as condomless sex

^d^transaction sex is defined as having sex with a man in exchange for food, clothes, cosmetics, transportation, and items for children, and other items

^e^Center for Epidemiologic Studies Depression Scale is the sum of 10 items (CESD-10) with a range of 0 to 30. CESD-10 score ≥ 10 indicates the likelihood of depressive symptoms

### Trust in the PrEP provider and PrEP adherence

**Fig. 1:**
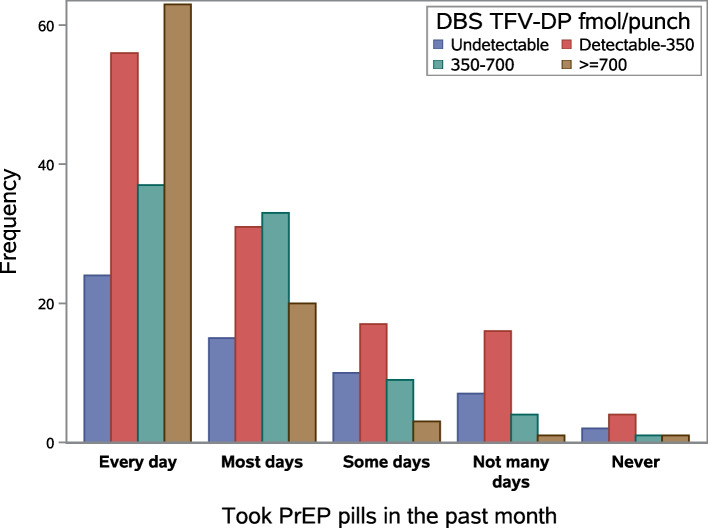
The frequency of dried blood spot tenofovir-diphosphate (biomarker) concentration by patient-reported pre-exposure prophylaxis (PrEP) adherence

Patient-reported adherence presented by biomarker DBS TFV-DP concentration is shown in Fig. 1. Among the participants who reported high adherence, 30% had high DBS TFV-DP (≥ 700 fmol/punch) concentrations, and among those who reported low adherence, 75% had low DBS TFV-DP (< 350 fmol/punch) concentrations.

Participants responded with a high level of agreement for the three indicators of trust in PrEP providers: “I have a strong, trusting relationship with the study staff” was 86%; “I let the study staff know if I miss doses of my PrEP” was 78%; and “I know how to contact the study doctor or nurse if I have problems or questions about PrEP” was 85%.

AGYW who reported strong, trusting relationships with their PrEP providers (OR 3.72, 95% CI 1.20–11.51, *p* = 0.02) and who knew who to contact for questions and problems about PrEP (OR 2.94, 95% CI 1.05–8.29, *p* = 0.04) had significantly increased odds of being concordant adherent than discordant non-adherent (Table 2). AGYW, who indicated that they would let study staff know if PrEP pills were missed (OR 2.04, 95% CI 0.93–4.51, *p* = 0.08), were marginally more likely to be concordant adherent than discordant non-adherent. In the multivariable logistic regression analysis, which included all three trust indicators, trusting relationships with the PrEP providers (aOR 3.72, 95% CI 1.20–11.51, *p* = 0.02) remained an independent predictor of concordance. When comparing concordant non-adherent to discordant non-adherent, none of the three trust indicators was significant. The results of the univariable sensitivity analysis, which compared high (TFV-DP ≥ 700 fmol/punch) adherence to *no-to-low* (undetectable TFV-DP) adherence, were consistent with the main analysis but did not achieve statistical significance.

**Table 2:** Associations between provider characteristics and PrEP adherence

Provider characteristics	Outcome	OR (95% CI)	*P*-value	AOR (95% CI)	*P*-value
**Trusting relationship** (agree vs. disagree)	Concordant adherent	3.72 (1.20, 11.51)	0.02	3.72 (1.20, 11.51)^a^	0.02
	Concordant non-adherent	0.81 (0.34, 1.92)	0.64		
	Discordant non-adherent	Reference		Reference	
**Let study staff know if missed pills** (agree vs. disagree)	Concordant adherent	2.04 (0.93, 4.51)	0.08		
	Concordant non-adherent	1.87 (0.81, 4.32)	0.14		
	Discordant non-adherent	Reference			
**Know who to contact for questions/problem about PrEP** (agree vs. disagree)	Concordant adherent	2.94 (1.05, 8.29)	0.04		
	Concordant non-adherent	1.37 (0.54, 3.52)	0.51		
	Discordant non-adherent	Reference			

*PrEP* Pre-exposure prophylaxis

^a^This odds ratio is of the three trust characteristics together for concordant adherent versus discordant non-adherent

**Fig. 2:**
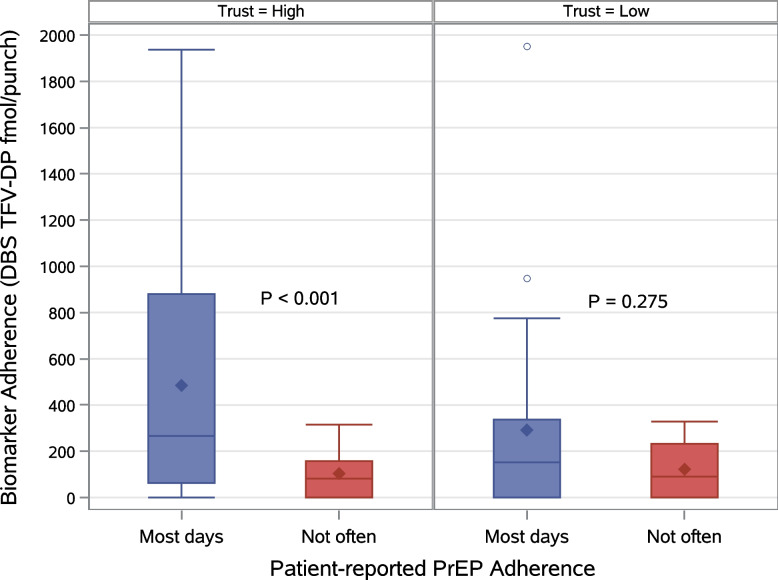
Dried blood spot tenofovir-diphosphate (DBS TFV-DP) concentrations by high and low levels of provider trust and by patient-reported adherence. Patient adherence responses: every day and most days are defined as ‘most days’; some days and not many days responses are defined as ‘not often’

The comparison of PrEP adherence biomarker concentration as a continuous measure was consistent with the primary analysis; AGYW who had *high trust* in the provider had significantly higher (*p* < 0.001) mean concentration of DBS TFV-DP if the patient’s self-reported adherence was ‘most days’ (485 fmol/punch) than ‘not often’ (104 fmol/punch). In contrast, AGYW, who reported *low trust* in the provider, had a non-significant (*p* = 0.275) mean difference in DBS TFV-DP concentration with 292 fmol/punch for ‘most days’ and 122 fmol/punch for ‘not often’ (Fig. 2).

## Discussion

This study provides empirical evidence that AGYW from Africa who had a trusting relationship with their PrEP providers were more likely to have congruence between patients’ self-reported adherence and concentrations of DBS TFV-DP than those without a trusting relationship. The finding is important because to increase PrEP adherence, providers must understand if patients are taking the medication. Since it is expensive and burdensome to collect biomarkers and self-report is easy, inexpensive, and presents little burden to patients, if self-reported adherence is accurate, then with this information, providers can deliver additional support or interventions to those who report low adherence.

We found about one-third of participants were discordant non-adherent, which is comparable to the SEARCH population-based universal PrEP access study in East Africa [41]. There are likely multiple reasons for discordant non-adherence. Even though AGYW who participated in the study were told that they could stop and start PrEP, they may have perceived that not adhering as prescribed would disappoint the study team and were inclined to report higher adherence than those not participating in a study [42]. The participants may also not have fully understood that PrEP providers respected their self-autonomy to choose how often to take PrEP pills, depending on changing ‘seasons’ of HIV acquisition likelihood (i.e., between relationships, the partner was away, or partner living with stable low viral load) [42–47]. Moreover, AGYW may have taken PrEP pills on an event-driven basis during the time of possible HIV exposure and subsequently reported higher adherence. The World Health Organization has recommended an event-driven 2 + 1 + 1 oral PrEP strategy (two pills taken between 2 and 24 h before sex, then a third pill 24 h after the first two pills, and a fourth pill 48 h after the first two pills) for MSM [48]. Data about the efficacy of event-driven PrEP dosing are not available for women and were not recommended in HPTN 082, although women may still have taken PrEP episodically. Since DBS drug concentration is a cumulative measure of pill taking during the prior 1–2 months, it cannot differentiate how well AGYW adhered during the occasions of possible HIV exposure in the past month; this may at least partly explain discordant non-adherence. On the other hand, due partly to adolescents’ cognitive-developmental stage and the overall challenges in knowing whether one’s partner is likely to be living with HIV, AGYW may not have accurately perceived their HIV acquisition likelihood [49–51]. However, this study signaled greater odds of concordant adherence among those who perceived a greater likelihood HIV acquisition (*p* = 0.07). This is consistent with findings of increased PrEP adherence among AGYW [52]. Given only four new HIV diagnoses were observed in the HPTN 082 study over a 1-year period [8], future research is needed to assess if AGYW are following an event-driven (i.e., on-demand) adherence strategy but do not feel empowered to communicate actual adherence patterns to their provider and the reasons for not adhering to taking daily PrEP pills as prescribed by the provider.

There are a few limitations to our study. The ‘trust’ indicator questions used in the paper were collected at month three and not from a scale validated for this population and we evaluated the three questions separately, which may limit validity. Therefore, developing developmentally appropriate trust questionnaire for AGYW will likely lead to more accurately capturing factors that affect having a trusting relationship with the provider. For example, the components of the Sandford Trust in Physician Scale includes questions about quality of care, maintaining personal information confidential, treating participants nonjudgmentally, and prioritizing participant needs over research goals [53].

The provider trust indicators collected in the HPTN 082 trial did not allow us to discern trust issues stemming from encounters with clinicians, other staff, or the healthcare system. Further, the study did not collect data on which aspects of interactions with providers and staff contributed to a low level of trust. However, all three *trust* indicators signaled high trust in the providers. Finally, adherence goal setting was not captured at each visit to reflect the dynamic nature of the AGYW’s sexual partnerships, which may be better aligned with patient-reported adherence. Goal setting recognizes AGYW’s self-autonomy, which can facilitate open communication with providers, including identifying barriers such as depression experienced by AGYW [35]. To build trust, PrEP providers must treat AGYW nonjudgmentally for their choices, including PrEP use and sexual behavior [21, 54]. A greater understanding of trust factors is essential to create effective tools that improve patient-provider interactions.

## Conclusions

Our study confirms that trust in the PrEP provider increases concordance between patient-reported PrEP adherence and biomarker concentration of PrEP for AGYW suggesting that when a patient trusts their provider, patient-reported adherence is an adequate adherence measure. With accurate knowledge of PrEP adherence, providers can deliver additional support or interventions as needed to increase the daily medication. Therefore, education and training to build trusting relationships between providers and AGYW may lead to more accurate reporting of PrEP adherence, and in-turn greater participation of AGYW in formulating an HIV prevention plan.

## Data Availability

The data underlying the results presented in the study are available from SCHARP data management center, HPTN- data-access@scharp.org. Analysis code may be shared upon request to Geetha Beauchamp (g3beauchamp@gmail.com).

## Abbreviations

AGYW: Adolescent girls and young women
ART: Anti-retroviral therapy
CASI: Computer-assisted self-interview
CESD-10: Center for Epidemiologic Studies Depression Scale
DBS: Dried blood spot
DP: Diphosphate
MSM: Men who have sex with men
TFV: Tenofovir
SOC: Standard of care
SMS: Short message service
WHO: World Health Organization
